# Inter-patient ECG heartbeat classification for arrhythmia classification: a new approach of multi-layer perceptron with weight capsule and sequence-to-sequence combination

**DOI:** 10.3389/fphys.2023.1247587

**Published:** 2023-09-28

**Authors:** Chenchen Zhou, Xiangkui Li, Fan Feng, Jian Zhang, He Lyu, Weixuan Wu, Xuezhi Tang, Bin Luo, Dong Li, Wei Xiang, Dengju Yao

**Affiliations:** ^1^ Key Laboratory of Electronic and Information Engineering, State Ethnic Affairs Commission, Southwest Minzu University, Chengdu, China; ^2^ Guangxi Key Laboratory of Digital Infrastructure, Guangxi Information Center, Nanning, China; ^3^ School of Computer Science and Technology, Harbin University of Science and Technology, Harbin, China; ^4^ West China Biomedical Big Data Center, West China Hospital, Sichuan University, Chengdu, China; ^5^ Sichuan Huhui Software Co., Ltd., Mianyang, China; ^6^ Med-X Center for Informatics, Sichuan University, Chengdu, China

**Keywords:** arrhythmia classification, multilayer perceptron, weight capsule, MIT-BIH, deep learning

## Abstract

**Objective:** The objective of this research is to construct a method to alleviate the problem of sample imbalance in classification, especially for arrhythmia classification. This approach can improve the performance of the model without using data enhancement.

**Methods:** In this study, we have developed a new Multi-layer Perceptron (MLP) block and have used a Weight Capsule (WCapsule) network with MLP combined with sequence-to-sequence (Seq2Seq) network to classify arrhythmias. Our work is based on the MIT-BIH arrhythmia database, the original electrocardiogram (ECG) data is classified according to the criteria recommended by the American Association for Medical Instrumentation (AAMI). Also, our method’s performance is further evaluated.

**Results:** The proposed model is evaluated using the inter-patient paradigm. Our proposed method shows an accuracy (ACC) of 99.88% under sample imbalance. For Class N, sensitivity (SEN) is 99.79%, positive predictive value (PPV) is 99.90%, and specificity (SPEC) is 99.19%. For Class S, SEN is 97.66%, PPV is 96.14%, and SPEC is 99.85%. For Class V, SEN is 99.97%, PPV is 99.07%, and SPEC is 99.94%. For Class F, SEN is 97.94%, PPV is 98.70%, and SPEC is 99.99%. When using only half of the training sample, our method shows that the SEN of Class N and V is 0.97% and 5.27% higher than the traditional machine learning algorithm.

**Conclusion:** The proposed method combines MLP, weight capsule network with Seq2seq network, effectively addresses the problem of sample imbalance in arrhythmia classification, and produces good performance. Our method also shows promising potential in less samples.

## 1 Introduction

Electrocardiogram plays an important role in the clinical diagnosis and treatment of cardiovascular diseases, but traditional methods only rely on professional physicians to analyze the electrocardiogram, which is not only time-consuming, but also requires high professional knowledge of doctors. Therefore, many researchers have been working on computer-aided diagnosis to improve the efficiency of ECG analysis (Luz et al., 2016; Ebrahimi et al., 2020).

Traditional machine learning arrhythmia classification methods rely on well-designed feature extraction methods and classification models to show acceptable arrhythmia classification performance. Compared with traditional machine learning algorithms, which require complex feature extraction, deep learning end-to-end ECG classification has attracted more and more attention (Parvaneh et al., 2019; Murat et al., 2020). Among them, convolutional neural network (CNN) and recursive neural network (Hochreiter and Schmidhuber, 1997; Sathasivam, 2008; Wu et al., 2016; Kim et al., 2017; Song et al., 2017) have been widely used in ECG classification (Oh et al., 2018; Petmezas et al., 2021). A hybrid solution has been found that combines representational learning with continuous learning to create powerful input data and thereby improve the performance of the model, which makes the combined approach of CNN and recurrent neural networks available for arrhythmia detection (Tan et al., 2018; Yildirim et al., 2019).

Although the combined method of convolutional network and cyclic neural network has improved the classification performance of arrhythmia, it is susceptible to the influence of sample imbalance, so its performance in the class with a small sample size is not ideal. At present, sampling and weighting are the main methods used by deep learning to solve the problem of sample imbalance in beat classification (Mousavi and Afghah, 2019; He et al., 2020; Jiang et al., 2020). Mousavi and Afghah (2019) combined convolutional neural networks with the Sequence to Sequence (Seq2Seq) model and integrated several classes of oversampling techniques to achieve excellent results in arrhythmia classification. Many studies have also adopted data enhancement methods such as Synthetic Minority Oversampling Technique (SMOTE). However, the SMOTE method may be skewed when producing a few types of samples.

To this end, in the past we used the method of combining weight capsules (Li et al., 2022) with Seq2Seq to solve the sample imbalance problem. The weight capsule network is an optimization of the capsule network, which alleviates the saturation of the compression function of the capsule network and the problem of considering the probability of the output vector in the dynamic routing. Capsule network is a new choice proposed by Sabour et al. (2017) to solve the problems of translation invariance and information loss in pooling operations of deep convolutional networks. Based on this promising work, Butun et al. (2020) proposed a one-dimensional capsule network. The method is used to diagnose coronary artery disease from the original ECG, and the best performance is achieved in the 2s ECG segment. Neela and Namburu (2021) converted ECG signals into spectral signals, and then used capsule network to classify ECG, and realized accurate classification of ECG. Jayasekara et al. (2019) built a time capsule to learn from the sequence data, and the model was able to classify ECG rare beats with only a few training samples. However, the weight capsule network only relies on the input to obtain the heartbeat characteristics, the weight capsule belt information is limited, and the model performance has room for improvement.

Multi-layer Perceptron (MLP) is a forward structured artificial neural network ANN. MLP is mainly used as classifier or feature extraction in arrhythmia classification, and has achieved good performance (Hsiao et al., 2022; Sivapalan et al., 2022). Recently, due to the success of deep learning, MLP has gained attention again (Lian et al., 2021; Liu et al., 2021; Zhao et al., 2021; Tang et al., 2022; Tang et al., 2022; Hou et al., 2022; Valanarasu and Patel, 2022; Yu et al., 2022). Existing studies have found that (Tolstikhin et al., 2021), compared with using convolutional neural networks for feature extraction, MLP can not only combine the information between different channels, but also the information of different spatial locations.

Capsule network has great potential for feature extraction and classification of time series data. But at the same time, the common capsule network still has some problems. Some scholars have put forward the capsule variable model successively and achieved good results (Wang and Liu, 2018; Choi et al., 2019), but their model fails to alleviate the saturation of compression function and consider the probability of the existence of output vector in dynamic routing. To alleviate these problems, we proposed Sigmoid-squash (S-S) compression function and weight capsule (WCapsule) model (Li et al., 2022). The weight capsule can better extract the features of time series data for classification. In addition, in order to adapt to variable-length sequences and better improve the performance of the model, it can be combined with Seq2seq.

This paper aims to alleviate the problem of sample imbalance in arrhythmia classification and construct an accurate and effective cardiac beat classification method without SMOTE. Inspired by the existing work (Li et al., 2022), we propose a new and effective arrhythmia classification method named MWCapsuleNet, which uses a weight capsule network with MLP to extract the characteristics of heartbeat beats. Our contributions are as follows:

(1) We propose an arrhythmia classification method combining MWCapsuleNet and Seq2seq, which not only improves the performance of the algorithm model in arrhythmia classification, but also provides a new scheme for alleviating sample imbalance.
(2) In order to better exploit the potential of weight capsules, we propose an improved MLP block structure and construct a weight capsule network with MLP. As far as we know, this is the first time that MLP and weight capsule network have been combined and have been applied to arrhythmia classification, which adds a new idea for further exploring the potential of weight capsules.

**FIGURE 1:**
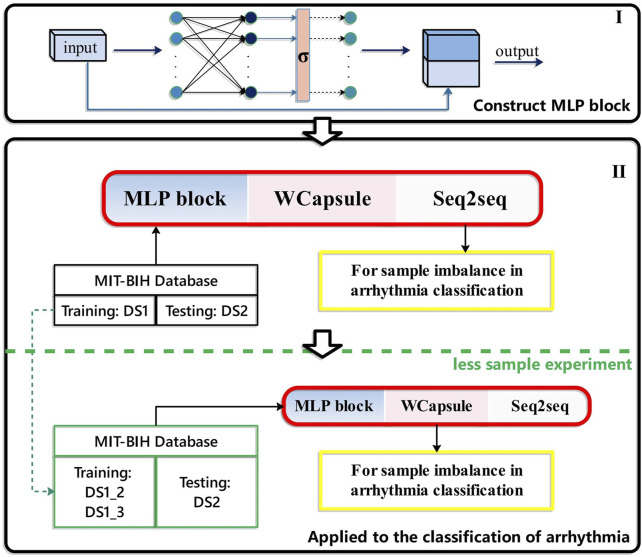
Study design. I, Construct MLP block; II, Applied to the classification of arrhythmia and less sample experiment.

The design process of this study is shown in Figure 1.

## 2 Material and methods

### 2.1 Dataset

In this work, we use the publicly accessible MIT-Beth Israel Hospital (MIT-BIH) arrhythmia database (Moody and Mark, 2001) to evaluate the performance of our proposed approach. The MIT-BIH dataset contains 48 ECG records, each containing a two-channel record of about 30 min. Two cardiologists annotated each note. In most recordings, one channel is the Modified Limb Lead II (MLII), obtained by placing electrodes on the chest, which is standard practice for hologram recordings, and the other is usually V1 (sometimes V2, V4, or V5, depending on the subject). Usually, the lead II is used to detect heartbeats in the literature (Mousavi and Afghah, 2019; Li et al., 2022; Wu et al., 2022; Xu et al., 2022; Zhu et al., 2022). Similarly, here in all experiments, we have applied ECG lead II. The raw ECG data were classified according to the AAMI recommended standard (Association for the Advancement of Medical Instrumentation et al., 1998), and the classification and description are shown in Supplementary Table S1.

Based on the recommendations of existing studies (De Chazal et al., 2004), we evaluate the proposed model using an interpatient model. The training and test sets are constructed from different patient sample data. By using this classification method, the inclusion of heartbeat records from the same patient in both the training and test sets is avoided, allowing the classification model to be fairly compared with other existing studies.

The interpatient assessment model divides the MIT-BIH database into two groups of records: DS1 = {101, 106, 108, 109, 112, 114, 115, 116, 118, 119, 122, 124, 201, 203, 205, 207, 208, 209, 215, 220, 223, 230} and DS2 = {100, 103, 105, 111, 113, 117, 121, 123, 200, 202, 210, 212, 213, 214, 219, 221, 222, 228, 231, 232, 233, 234}. In addition, in order to explore the potential of this work, we divide DS1 (training set) into DS1_2 = {101, 106, 108, 109, 112, 114, 115, 116, 118, 119, 122}, DS1_3 = {124, 201, 203, 205, 207, 208, 209, 215, 220, 223, 230}. Detailed data sample distribution is shown in Supplementary Table S2. We use DS1, DS1_2 and DS1-3 to train classification models, and use DS2 to evaluate the training models.

### 2.2 Methods

Figure 1 shows the overall flow chart of the combined MLP and WCapsule applied to arrhythmia classification, including: (1) The improved MLP structure, (2) The arrhythmia classification experiment based on the MWCapsuleNet + seq2seq model and the low-sample arrhythmia classification experiment.

#### 2.2.1 MLP block

**FIGURE 2:**
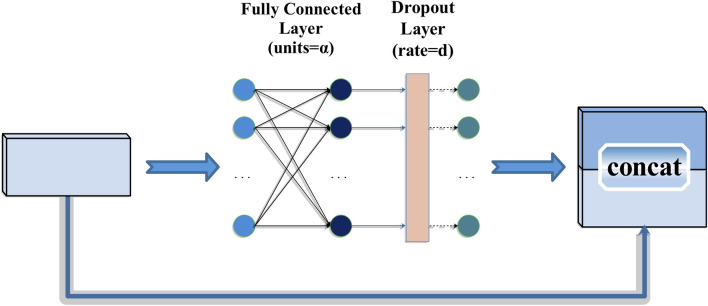
Structure of MLP block.

The MLP block structure designed in this work is shown in Figure 2, which mainly consists of two parts: MLP layer and skip layer connection. In the MLP layer, it has a fully connected layer after feature extraction of the fully connected layer. Secondly, we add the dropout layer to prevent overfitting. Finally, we extract the global feature vector of the heartbeat data and then merge it with the original input data as the output of MLP block.

In Figure 2, the input data of MLP is divided into chunks (patches), and the size of each patch is *p* × *p*, which can be divided into $S=\frac{HW}{p^{2}}$ patches in total. Flatten each patch with the size of *p* × *p*, that is, expand into a one-dimensional vector, and obtain a vector with length *p* ^2^. S of these vectors are put together to form a tensor of dimension (*S*, *p* ^2^), and the tensor is mapped linearly to the size of the second dimension C, which is called hidden dimension. Then we have a tensor with dimensions of (*S*, *C*) = *patches* × *channels*. It’s made up of S 1 × *C* vectors. This tensor *X* ∈ *R* ^(*S*×*C*)^ is the real input for subsequent MLP models. In this work, 10 × 28 after reshaping can be viewed as *patches* × *channels*. The MLP model of this work can be expressed as:

$$U_{ij}=Dropout\left(FC_{1}\left(Input_{i,j}\right)\right)\;for\;i=1\;\ldots S;\;j=1\;\ldots C$$ (1)

$$Output=concat\left(U_{ij},Input\right)$$ (2)

here *FC*_1_ means the first fully connected layer. Here *Dropout* means dropout layer.

Therefore, in the MLP block of the final model, the number of neuron units in the fully connected layer was set as 10. After several experiments, we finally found that the model achieves the best performance when the dropout rate is set to 0.8. Some of the experimental results are shown in Supplementary Material Supplementary Table S7.

#### 2.2.2 Arrhythmia classification method based on MLP+WCapsule+Seq2seq

MLP, weight capsule network and Seq2seq model constitute the model proposed in this work. The weight capsule network is the inheritance and development of the capsule network, and has shown satisfactory potential in alleviating the problem of sample imbalance in arrhythmia classification (Li et al., 2022). The Seq2Seq model is an important model in neural machine translation, and has shown close to human level in the application of (Johnson et al., 2017). Here, we use Recurrent Neural Network (RNN) Seq2Seq model and weight capsule network with MLP for arrhythmia classification task.

**FIGURE 3:**
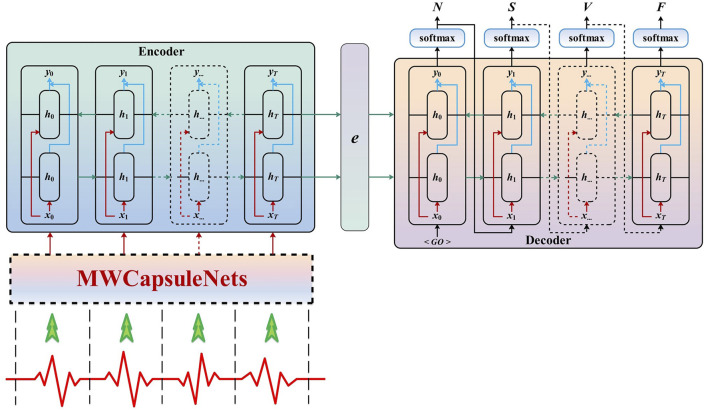
Construction of the algorithmic for arrhythmia classification.

**FIGURE 4:**
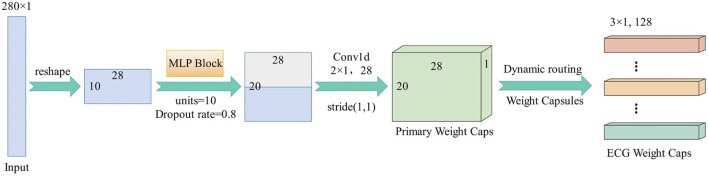
Flowchart of the proposed method.

Figure 3 shows the proposed framework for arrhythmia classification. The weight capsule network with MLP is composed of a layer of MLP block layer, a one-dimensional convolution layer and a weight capsule layer. The original data is a 280 × 1 time series signal after simple preprocessing. After being reshaped into 10 × 28, it is output as a 20 × 28 feature map through MLP block. This feature map first passes through the convolutional weight capsule layer, with single-channel one-dimensional convolution 28D weight capsules (i.e., each sovereign weight capsule contains 28 one-dimensional convolution units with 3 × 1 cores and step spacing of 1), and then activates the corrected linear unit (ReLU). The second layer (ECG Weight Caps) has 128 weight capsules with size of 3 × 1. Each capsule receives input from the weight capsule directly connected to it in the next layer. As a result, the initial weighted capsule output $\left({\dot{u}}_{i}\right)$ are passed into $\left({\dot{v}}_{j}\right)$ with equal probability $\left({\dot{c}}_{ij}\right)$ . After extracting the features of the maximum time dimension, ECG Weight Caps Layer performs the correction of *k* × 1 vector and outputs (e.g., here we set the output dimension to 128). Finally, the input sequence for each beat is associated with the vector *C* ∈ *R* ^*d*^. Figure 4 depicts the detailed network.

When the length of the capsule module is large, it will lead to the saturation problem of the compression function. In order to alleviate this problem, we proposed a new variant compression function S-S (Sigmoid-squash) (Li et al., 2022), as shown in Eq 3:

$$\dot{v_{j}}=\frac{g\left(h\left({\left\|{\dot{s}}_{j}\right\|}^{2}\right)\right)+\alpha \left\|{\dot{s}}_{j}\right\|}{1+\alpha \left\|{\dot{s}}_{j}\right\|+g\left(h\left({\left\|{\dot{s}}_{j}\right\|}^{2}\right)\right)}$$ (3)

*α* is a hyperparameter, ${\dot{v}}_{j}$ is the output vector, ${\dot{s}}_{j}$ is the input vector. Set *α* = 0.1.

The low-level weight capsule ${\dot{u}}_{i}$ is multiplied with the weight matrix ${\dot{W}}_{ij}$ to obtain the prediction vector ${\hat{\dot{u}}}_{j|i}$ .

$${\hat{\dot{u}}}_{j|i}={\dot{W}}_{ij}{\dot{u}}_{i}$$ (4)

The weighted capsule model is to multiply the weighted sum of the prediction vector ${\hat{\dot{u}}}_{j|i}$ and the weight coefficient ${\dot{c}}_{ij}$ with the weight *k*_*j*_ (Formula S4 in Supplementary Material). The results with weight selection are then output after S-S compression function. Therefore, except for the first layer, the input to the weight capsule ${\dot{s}}_{j}$ can be expressed as:

$${\dot{s}}_{j}=k_{j}\sum_{i}{\dot{c}}_{ij}{\hat{\dot{u}}}_{j|i}$$ (5)

Here, ${\dot{c}}_{ij}$ is the coupling coefficient determined by the iterative process of dynamic routing.

$${\dot{c}}_{ij}=\frac{\exp\left({\dot{b}}_{ij}*f_{ij}\right)}{\sum_{k}\exp\left({\dot{b}}_{ij}*f_{ij}\right)}$$ (6)

Where ${\dot{b}}_{ij}$ is the connection coefficient of two weight capsules. The weight *f*_*ij*_ (Formula S8 in Supplementary Material) can reduce the correlation between the predicted weight capsule mold length and the two weight capsules.

Based on the weighted capsule model, we updated the dynamic routing between the capsules. The main process of dynamic routing algorithm and pseudo-code for dynamic routing between weight capsules are shown in Supplementary Material Section 2.

The Seq2seq model in this work is the same as that used in the past work, where the encoder encodes the input sequence and the decoder calculates the category of each beat of the input sequence. The encoder is actually composed of long-and-short-term memory (LSTM) units, which are also known as many-to-one LSTM. As shown in Figure 3. Instead of the standard LSTM (i.e., RNN), we use bidirectional recursive neural network (BiRNN) units in the network architecture. Standard RNNS are unidirectional; they are limited to using previous input states. BiRNN, which can process data forward and backward, and the current state can access both previous and future input information. BiRNN consists of a forward network and a backward network. The input sequence is fed in normal time order, the forward network is fed *t* = 1, …, *T*, and the backward network is fed in reverse time order *t* = *T*, …, 1. Finally, the weighted sum of the two network outputs is calculated as the output of the BiRNN. The mechanism can be expressed as follows:

$${\vec{h}}_{t}=\tanh\left(\vec{W}x_{t}+\vec{V}{\vec{h}}_{t-1}+\vec{b}\right)$$ (7)

$${\overset{⃖}{h}}_{t}=\tanh\left(\overset{⃖}{W}x_{t}+\overset{⃖}{V}{\overset{⃖}{h}}_{t+1}+\overset{⃖}{b}\right)$$ (8)

$$y_{t}=\left(U\left[{\vec{h}}_{t};{\overset{⃖}{h}}_{t}\right]+b_{y}\right)$$ (9)

Here, $\left({\vec{h}}_{t},\vec{b}\right)$ is the hidden state and deviation of the forward network, and $\left({\overset{⃖}{h}}_{t},\overset{⃖}{b}\right)$ is the hidden state and deviation of the backward network. *x*_*t*_ and *y*_*t*_ are the input and output of BiRNN, respectively. The decoder is used to generate the target sequence beat by beat. Like an encoder, the building block of a decoder is an LSTM, but it is a many-to-many LSTM. The decoder gets a new representation of the input sequence generated by the encoder to initialize its hidden state. It also shifts the same given target by one and takes a special feature vector <GO> as input. It is important to note that the input (the shifted target) is only used during the training phase, not the test phase. Then, using softmax on the output of the LSTM, convert it to a probability *p* ∈ *R* ^*C*^, where C represents the number of categories (that is, the heartbeat type) and each element of *p* represents the probability of each class in the category.

#### 2.2.3 Comparative experimental model

In order to better evaluate our approach, we compared different heartbeat feature extraction models under the same equipment and environment and the same Seq2seq classification model as in this work. These model structures are all different combinations of MLP blocks, convolutional layers, ordinary capsules and weight capsules. It mainly includes: Baseline (Mousavi and Afghah, 2019), MLP block, McNets(MLP block + conv), M-Baseline(MLP block + Baseline), CapsuleNets(conv + Capsule), MCapsuleNets(MLP block + Capsule), WCapsuleNets(conv + WCapsule), and CWCapsuleNets(conv block + WCapsule).

The structure diagram for each specific model is shown in Supplementary Material S3.

## 3 Experiment and result

### 3.1 Data preprocessing

The input of the model in this work is a series of heart beats. In order to extract heart beats from a given ECG signal, according to the recommendations of existing studies (Mousavi and Afghah, 2019), we use the same method to preprocess the original data, which includes the following simple steps:

(1) Normalize the given ECG signal to be between 0 and 1;
(2) Search the R-wave set of ECG by the corresponding annotation file in MIT-BIH arrhythmia database;
(3) Segment the continuous ECG signal into a series of heartbeats according to the extracted R-wave, and assign a label to each heartbeat according to the annotation file;
(4) Resize each heartbeat to a predefined fixed length (280 samples).

These preprocessing steps for beat extraction are very simple and do not involve any form of filtering or noise removal methods. The source data preprocessing code used in this work is available here.

### 3.2 Experimental parameter

We build the model based on TensorFlow 2.4, using Adam optimizer and cross entropy loss function, with a learning rate of 0.001 and a maximum training period of 500. The initial LSTM hiding and cell state of the Seq2Seq model are set to 0. The drop rate of MLP is set to 0.8.

In order to compare with existing studies, four indexes are mainly used in the evaluation: sensitivity (SEN), specificity (SPEC), positive predictive value (PPV) and accuracy (ACC).

$$SEN=\frac{TP}{TP+FN}$$ (10)

$$PPV=\frac{TP}{TP+FP}$$ (11)

$$SPEC=\frac{TN}{TN+FP}$$ (12)

$$ACC=\frac{TP+TN}{TP+TN+FN+FP}$$ (13)

Here, TP (true positive), TN (true negative), FP (false positive), and FN (false negative) represent the number of heartbeats correctly labeled, correctly identified as non-corresponding heartbeats, incorrectly labeled, and not identified as expected heartbeats, respectively.

### 3.3 Result

Because the sample size of class Q is too small, and the reference methods did not take it into account. Therefore, we do not list it in the comparison results table. When comparing with a wide range of earlier publications, we use the results published in their original papers as a reference. The differences calculated in this work are all differences in percentage points.

In order to evaluate the effectiveness of our innovative elements, we evaluate the model performance using DS1 as the training set and DS2 as the test set. It is worth noting that the number of parameters of the model proposed in this work is less than that of the baseline model (252980 < 357154).

#### 3.3.1 Sample imbalance

Three-classification task: As shown in Table 1, our method obtains the best results in 8 out of 10 evaluation indicators, the overall indicator performance is higher than 94.5%, and the maximum difference between other indicators and the optimal index is 0.12%. In class S, where the training samples are less than the test samples, after MLP block module is added to the weight capsule network, SEN = 98.75%, PPV = 94.82%, SEN and PPV are increased by 5.21% and 2.6% respectively. After adding MLP block to the capsule network, 80% of the indicators have been improved. Using only the MLP block, although the PPV of the S class is only 83.01%, its overall performance is close to that of the baseline. In addition, CWCapsuleNets using convolution block performs poorly in class S (SEN = 80.01%, PPV = 74.27%). Compared with the research using DS1 training model, as shown in Table 3, in the three-classification task, Jiang et al. (2020) obtained the best performance with SMOTE. Compared with that, our model obtains 98.75% SEN in class S without SMOTE, 2.06% higher than them.

**TABLE 1:** Inter-patient paradigm: The performance of the proposed heartbeat classifier compared with other comparison models, considering DS2 as test dataset based on the MIT-BIH arrhythmia database for the considered groups: N, S, V. SMOTE is not used in this experiment.

Model	ACC	N			S			V		
		SEN	SPEC	PPV	SEN	SPEC	PPV	SEN	SPEC	PPV
Baseline	99.37	99.69	93.89	99.31	82.03	99.78	93.54	**100**	99.88	98.34
MLP + Seq2seq	99.39	99.06	99.34	99.92	97.96	99.23	83.01	**100**	99.88	98.31
McNets + Seq2seq	99.52	99.57	97.06	99.66	91.94	99.65	91.05	99.50	99.91	98.76
M-Baseline	99.41	99.75	93.91	99.31	83.19	99.74	92.40	99.50	**100**	**100**
CapsuleNets + Seq2seq	99.40	99.32	98.55	99.83	96.02	99.22	82.61	99.00	**100**	**100**
MCapsuleNets + Seq2seq	99.71	99.63	99.35	99.93	97.44	99.72	93.13	**100**	99.90	98.59
CWCapsuleNets + Seq2seq	98.78	98.81	92.93	99.19	80.01	98.93	74.27	99.81	99.93	98.98
WCapsuleNets + Seq2seq	99.64	99.67	97.74	99.74	93.54	99.70	92.22	**100**	99.99	99.88
MWCapsuleNets + Seq2seq	**99.83**	**99.78**	**99.62**	**99.96**	**98.75**	**99.79**	**94.82**	**100**	99.99	99.88
Baseline*	98.54	98.88	91.47	99.02	68.41	99.46	83.12	**100**	99.15	89.18
MLP + Seq2seq*	97.12	95.69	98.26	99.79	95.37	95.89	47.35	95.77	99.80	97.16
McNets + Seq2seq*	97.96	96.75	**99.23**	**99.91**	96.77	97.10	56.22	99.60	99.80	97.15
M-Baseline*	97.76	96.89	96.12	99.54	87.96	98.39	67.91	98.13	98.49	81.95
CapsuleNets + Seq2seq*	98.30	97.64	96.32	99.57	89.11	97.96	62.85	99.69	99.78	96.95
MCapsuleNets + Seq2seq*	96.67	94.64	98.61	99.83	96.19	95.30	44.25	99.50	99.65	95.25
CWCapsuleNets + Seq2seq*	99.09	98.60	99.11	99.90	**97.33**	98.70	74.37	99.75	99.97	99.57
WCapsuleNets + Seq2seq*	98.48	99.01	87.10	98.54	62.85	99.12	73.50	**100**	99.88	98.34
MWCapsuleNets + Seq2seq*	**99.60**	**99.81**	96.59	99.61	90.63	**99.75**	**93.27**	98.91	**100**	**100**

* indicates that DS1_3 is used as the training set, and the values in bold are the highest value in this metric.

Without * means that DS1 is used as the training set, and the values in bold and underlined are the highest value in this metric.

Five-classification task: In order to further evaluate our method, we increase the difficulty of classification. As shown in Table 2, our model obtains most of the highest indicators, accounting for almost 70% of all indicators, and the overall performance is higher than 97%. We can see from Table 2 that only the three models that adopt weight capsules can make the indexes of class S and class F reach more than 90%. Among the three models that also adopt weight capsules, our model can obtain the best performance. Similar to the performance improvement of capsule network after adding MLP block in the three-classification task in Table 1, the performance of 11 indicators in the five-classification task is also improved. While only using MLP block, although it surprisingly obtains the optimal values of five indicators, and the overall performance ranks fourth among the seven comparison models, the SEN of S class is only 82.30%, while the SEN of our method is 97.66%. In the five-classification task, as shown in Table 4, although Li et al. (2022) obtains the best performance of the existing literature, the PPV in class S is 92.23%, and the PPV of our method in class S is 3.91% higher than theirs.

**TABLE 2:** Inter-patient paradigm: The performance of the proposed heartbeat classifier compared with other comparison models, considering DS2 as test dataset based on the MIT-BIH arrhythmia database for the considered groups: N, S, V, F. SMOTE is not used in this experiment.

Model	ACC	N			S			V			F		
		SEN	SPEC	PPV	SEN	SPEC	PPV	SEN	SPEC	PPV	SEN	SPEC	PPV
Baseline	98.92	99.06	83.28	97.97	54.74	99.18	71.89	**99.97**	99.96	66.27	77.58	99.96	93.77
MLP + Seq2seq	99.59	99.69	93.53	99.21	82.30	99.70	91.24	**99.97**	**99.98**	**99.69**	90.21	**100**	**100**
McNets + Seq2seq	99.57	99.22	98.05	99.76	91.45	99.37	84.80	**99.97**	99.88	98.29	93.92	99.86	83.92
M-Baseline	99.52	99.69	91.86	99.00	76.74	99.90	96.71	**99.97**	99.89	98.44	94.07	99.89	87.11
CapsuleNets + Seq2seq	98.87	98.72	86.64	98.36	64.87	98.75	66.69	99.56	99.94	99.07	55.67	99.90	81.20
MCapsuleNets + Seq2seq	99.74	99.60	98.00	99.75	93.85	99.81	94.88	99.66	99.96	99.47	95.62	99.78	77.78
CWCapsuleNets + Seq2seq	99.76	99.62	98.16	99.77	93.79	99.67	91.64	**99.97**	99.94	99.10	96.65	99.97	95.66
WCapsuleNets + Seq2seq	99.81	99.76	98.47	99.81	95.26	99.77	94.03	99.63	99.96	99.38	94.07	99.96	94.81
MWCapsuleNets + Seq2seq	**99.88**	**99.79**	**99.19**	**99.90**	**97.66**	**99.85**	**96.14**	**99.97**	99.94	99.07	**97.94**	99.99	98.70
Baseline*	99.27	99.13	92.63	99.09	79.41	99.01	75.54	97.82	99.95	**99.30**	84.28	99.99	97.90
MLP + Seq2seq*	97.19	89.69	**99.12**	44.62	**92.50**	97.63	**99.69**	93.26	95.40	43.64	**99.97**	98.44	81.62
McNets + Seq2seq*	97.70	95.43	87.32	98.39	57.73	97.14	43.73	99.66	99.23	90.01	90.21	99.10	44.19
M-Baseline*	98.91	97.72	95.72	99.46	87.47	98.21	65.28	97.94	99.85	97.88	89.69	99.61	64.21
CapsuleNets + Seq2seq*	98.33	96.80	89.71	98.71	68.36	97.65	52.80	**100**	99.19	89.94	83.51	99.98	97.59
MCapsuleNets + Seq2seq*	97.80	94.33	97.22	**99.64**	91.29	94.99	41.18	98.72	99.78	96.84	97.16	99.84	83.04
CWCapsuleNets + Seq2seq*	99.53	99.47	94.91	99.37	83.39	99.51	86.79	99.94	99.88	98.35	91.94	99.96	94.92
WCapsuleNets + Seq2seq*	99.33	98.94	94.34	99.30	84.10	99.14	78.94	99.66	99.91	98.77	84.79	99.84	81.03
MWCapsuleNets + Seq2seq*	**99.75**	**99.95**	95.55	99.45	86.87	**99.91**	97.37	99.97	**99.94**	99.07	88.14	**100**	**100**

* indicates that DS1_3 is used as the training set, and the values in bold are the highest value in this metric.

Without * means that DS1 is used as the training set, and the values in bold and underlined are the highest value in this metric.

#### 3.3.2 Sample balance

To better evaluate the performance of MLP block and MWCapsuleNets in our innovative work, we augment the data with SMOTE method, as shown in Supplementary Material S4. In five-classification task, our method overall index is higher than 95%, and only three indicators do not achieve the highest. As can be seen in Supplementary Table S5, the top metrics are all from the model employing the weight capsule network, and the performance is further enhanced by adding the MLP block to the weight capsule model. As shown in Supplementary Table S4, in the three-classification task, the performance of models using the weight capsule network is above 94.5%. Our model has increased the SEN and PPV of Class S by 9.43% and 3.56%, respectively, compared with the baseline. However, in the three-classification task, performance of the models with MLP block or conv block decreases.

### 3.4 Model exploration

In order to better tap the potential of our method, we conduct less-sample exploration on the model. Because DS1_2 has a worse sample distribution than DS1 (as shown in Supplementary Table S2), the model does not work on DS1_2 (as shown in Supplementary Table S6). We mainly observe the results of using DS1_3 as the training set and DS2 as the test set. As shown in Table 2, our model has achieved the most balanced and excellent performance among the five-classification task, and half of the indexes have achieved the highest. Although MLP + Seq2seq has obtained the 4 highest indexes, its PPV of Class V is only 43.64%. It is 55.43% lower than our method (99.07%). It can be seen from Table 2 that the top three models with the highest overall performance all use weight capsules, with an overall performance higher than 78%. Meanwhile, the performance of MWCapsuleNets with MLP block is further improved compared with the weight capsule network. As shown in Table 1, compared with other model structures, in the three-classification task, all evaluation indicators of our model are higher than 90%, and we obtain 60% of the best indicators. As shown in Table 3, compared with the existing research, the overall performance of our method ranks third in the existing literature, and two indicators obtain the best level of the existing literature. As shown in Table 4, among the five-classification task, compared with existing literature, the overall performance of our method (training with DS1_3) is lower than Li et al. (2022) (training with DS1), but our method achieves the best level of existing literature in PPV of class S (97.37%), SEN of class N (99.95%), SEN of class V (99.97%), PPV and SPEC of F (100% & 100%).

**TABLE 3:** Inter-patient paradigm: Comparison of performance of the proposed heartbeat classifier against the state-of-the-art algorithms, considering DS1 as training dataset and DS2 as test dataset based on the MIT-BIH arrhythmia database for the considered groups: N, S, V.

Model	ACC	N			S			V		
		SEN	PPV	SPEC	SEN	PPV	SPEC	SEN	PPV	SPEC
Ours	99.83	99.78	**99.96**	**99.62**	**98.75**	94.82	99.79	**100**	99.88	99.99
Ours*	99.60	99.81	99.61	96.59	90.63	93.27	99.75	98.91	**100**	**100**
(Li et al., 2022)^*DL*^	99.67	99.59	99.91	99.25	96.62	91.16	99.64	**100**	98.90	99.60
(Kim et al., 2023)^*DL*^	89.10	91.40	97.70	-	49.30	26.60	-	91.40	65.70	
(Dias et al., 2021)^*ML*^	96.00	94.50	99.40	95.30	92.50	39.90	94.60	88.60	94.80	99.70
(Jiang et al., 2020)^*DL*^	**99.89**	**99.87**	99.84	98.56	95.69	97.06	**99.89**	99.98	99.89	99.97
(He et al., 2020)^*DL*^	95.10	97.50	97.60	-	83.80	59.40	-	80.40	90.20	-
(Niu et al., 2019)^*DL*^	96.40	98.90	97.40	-	76.50	76.60	-	85.70	94.10	-
(Mousavi and Afghah, 2019)^*DL*^	99.53	99.68	96.05	99.55	88.94	**99.72**	92.57	99.94	99.97	99.50

The values in bold and underlined are the highest value in this metric.

ML: using machine learning methods.

DL: using deep learning methods.

**TABLE 4:** Inter-patient paradigm: Comparison of performance of the proposed heartbeat classifier against the state-of-the-art algorithms, considering DS1 as training dataset and DS2 as test dataset based on the MIT-BIH arrhythmia database for the considered groups: N, S, V, F.

Model	ACC	N			S			V			F		
		SEN	PPV	SPEC	SEN	PPV	SPEC	SEN	PPV	SPEC	SEN	PPV	SPEC
Ours	**99.88**	99.79	**99.90**	99.19	97.66	96.14	99.85	**99.97**	99.07	99.94	**97.94**	98.70	99.99
Ours*	99.75	**99.95**	99.45	95.55	86.87	**97.37**	**99.91**	**99.97**	99.07	99.94	88.14	**100**	**100**
(Li et al., 2022)^*DL*^	99.85	99.66	99.97	**99.72**	**99.56**	92.23	99.68	**99.97**	**99.38**	**99.96**	93.81	**100**	**100**
(Zhu et al., 2022)^*DL*^	—	98.42	98.10	—	67.17	70.11	—	94.66	97.35	—	50.06	47.64	47.75
(Xu et al., 2022)^*DL*^	98.73	98.87	98.64	88.87	83.06	82.40	99.32	93.46	90.04	99.29	37.89	99.32	**100**
(Wang et al., 2022)^*ML*^	95.60	91.70	99.10	—	89.90	36.70	—	87.80	96.60	—	55.40	16.30	—
(Wu et al., 2022)^*DL*^	96.76	93.15	98.18	86.00	80.23	49.40	96.85	90.99	83.09	98.72	12.63	4.05	97.65
(Dias et al., 2021)^*ML*^	90.29	79.64	99.51	96.80	91.32	40.34	94.81	87,26	93.15	99.55	81.14	4.50	86.24
(Essa and Xie, 2021)^*DL*^	97.90	98.03	97.48	80.27	65.51	66.19	98.56	93.91	94.55	99.62	19.33	41.67	99.79
(Shi et al., 2020)^*DL*^	97.10	95.26	98.92	91.57	90.74	47.85	96.20	92.92	84.49	98.82	0.26	1.72	99.88
(Chen et al., 2020)^*DL*^	98.41	99.15	97.81	81.96	63.90	76.10	99.23	88.84	96.78	99.80	47.68	54.25	99.68
(Mondéjar-Guerra et al., 2019)^*ML*^	94.50	95.90	98.20	—	78.10	49.70	—	94.70	93.90	—	12.40	23.60	—
(Li and Zhou, 2016)^*ML*^	94.61	94.67	99.73	—	27.27	0.16	-	93.74	90.18	—	20.00	0.26	-
(Zhang et al., 2014)^*ML*^	88.94	98.98	98.98	—	79.06	35.98	-	85.48	92.75	—	93.81	13.73	—
(Ye et al., 2012)^*ML*^	94.57	88.51	97.54	82.33	60.80	52.34	97.72	81.49	61.38	96.46	19.59	2.50	94.00
(De Chazal et al., 2004)^*DL*^	86.20	87.10	99.20	—	76.00	38.50	—	80.30	86.60	—	89.40	8.60	—

The values in bold and underlined are the highest value in this metric.

* indicates that DS1_3 is used as the training set.

ML: using machine learning methods.

DL: using deep learning methods.

## 4 Discussion

In order to alleviate the problem of unbalanced distribution of heartbeat class samples in arrhythmia classification, this work innovatively proposes MWCapsuleNets + Seq2seq, which combines the improved MLP and weight capsule network. It not only has excellent performance on classes with a large sample size, but also obtains satisfactory performance on classes with a small sample size. The performance on less-samples is also expected.

Traditional machine learning arrhythmia classification methods achieve acceptable performance through preprocessing, segmentation, feature extraction and classification steps (De Chazal et al., 2004; Ye et al., 2012; Zhang et al., 2014; Li and Zhou, 2016; Mondéjar-Guerra et al., 2019; Wang et al., 2022). However, traditional machine learning arrhythmia classification methods need to rely on novel feature extraction methods and well-selected classifiers to improve the performance. However, our method is end-to-end, using MWCapsuleNets to automatically extract features and put the extracted features into the Seq2Seq model for classification. As shown in Table 4, compared with the best performance obtained by traditional machine learning methods, our method improves the PPV by 43.8% and 75.1% respectively in the S and F classes where it performs poorly, and the overall performance is more stable.

Compared with traditional machine learning algorithms, which require complex feature extraction, deep learning end-to-end ECG classification has attracted more and more attention from scholars (Hochreiter and Schmidhuber, 1997; LeCun et al., 1998; Sathasivam, 2008; Wu et al., 2016; Kim et al., 2017; Krizhevsky et al., 2017; Song et al., 2017; Oh et al., 2018; Tan et al., 2018; Attia et al., 2019; Hannun et al., 2019; Parvaneh et al., 2019; Yildirim et al., 2019; Murat et al., 2020; Ribeiro et al., 2020; Petmezas et al., 2021). As shown in Table 3 and Table 4, arrhythmia classification using deep learning methods such as convolutional network, U-Net, attention mechanism and LSTM has shown acceptable performance. However, due to the influence of sample imbalance, the expected performance has not been achieved. At present, sampling and weighting are the main methods used to solve the problem of sample imbalance in the classification of heartbeats (Mousavi and Afghah, 2019; He et al., 2020; Jiang et al., 2020). Capsule network has great potential for feature extraction and classification of time series data. As shown in Table 4, the method of weight capsule network combined with Seq2Seq (Li et al., 2022), effectively alleviates the sample imbalance problem by using the advantages of model structure. In the five-classification task, the best performance of the existing literature was obtained. Although the method of Li et al. was successful, it performed poorly in categories with small sample size. Their model did not fully meet expectations in S category’s PPV (92.23%) and F category’s SEN (93.81%). In order to further improve the performance of arrhythmia classification model, we proposed a novel weight capsule network with MLP combined with Seq2seq for arrhythmia classification on the basis of Li et al., and further enhanced the feature extraction capability of weight capsule network through MLP. Compared with the results before (Li et al., 2022), as shown in Table 3, our method achieved improvement in 10 evaluation indexes of the three-classification task, especially the SEN and PPV of class S increased by 2.13% and 2.66%, respectively. As shown in Table 4, in the five-classification task with increasing classification difficulty, the PPV of S and the SEN of F are improved by 3.91% and 4.31%, respectively while the SEN of S (97.66%) is 1.9% lower than the performance obtained before (Li et al., 2022). However, the overall performance index of our method ( > 96%) is better than that of Li et al. (2022) ( > 92%). As shown in Table 3, although some studies have achieved good performance using SMOTE method (Mousavi and Afghah, 2019; He et al., 2020; Jiang et al., 2020). But SMOTE method may have bias. Our method only relies on weight capsule network with MLP to enhance the acquisition of features without using oversampling (SMOTE, etc.) and weighted enhancement data, and achieve close or above the best (Jiang et al., 2020) of using the SMOTE method in this simple way. Moreover, our model has a smaller number of parameters than previous work (Mousavi and Afghah, 2019) (252980 < 357154). This shows that the combination of weight capsule network with MLP and Seq2Seq to alleviate sample imbalance is promising. At the same time, the clever combination of MLP and weight capsule network provides a new case for the progress of weight capsule network.

Previous studies have shown that capsules can classify rare ECG beats (Jayasekara et al., 2019), which has the potential to learn multiple sample sizes from a small sample size. Also, inspired by the good performance of our method on class S (training samples less than test samples), we designed less-sample experiments (sample size distribution is shown in Supplementary Material S1). As shown in Supplementary Table S2 the number of training samples of class S is less than the number of test samples (941 < 1836). In the less-sample experiments, we use DS1_2 and DS1_3 as the training set. Since the sample distribution of DS1_2 is more extreme, we mainly observe the results of training with DS1_3. (The results of training with DS1_2 are shown in Supplementary Material Supplementary Table S6.) As shown in Table 3, in the three-classification task, compared with the performance of our method training with full samples, there are three indicators better than that of full samples, and there are four indicators decreases less than 1%. However, SPEC of class N decreased by 3.03%, SEN of class S and V decreased by 8.12% and 1.09%, respectively. Compared with the existing studies, the overall performance index of our method ranked third in the existing literature (Jiang et al. (Jiang et al., 2020) obtained the optimal overall performance index by using SMOTE, and our previous work (Li et al., 2022) obtained the overall performance index by using full sample ranked second). As shown in Table 4, in the five-classification task, there are eight indicators the same as or better than that of full samples. Compared with the existing studies, the performance of our method ranked second in the existing literature, second only to the performance of (Li et al., 2022) with all samples. Considering that we use only half of the training samples, DS1_3 has less training sample than test sample for each category, such a result is acceptable. This also demonstrates the excellent feature extraction capability of our model.

In order to better alleviate the problem of sample imbalance in arrhythmia classification, we also consider other innovative elements. The results are shown in Table 1 and Table 2; Supplementary Table S4, S5. When training with the full sample, MLP is close to baseline. However, MLP needs to be combined with other networks to further exert the advantages of feature extraction when training with less sample. When combined with MLP block, MWCapsuleNets performs better than MCapsuleNets. Also, in combination with the same weight capsule network, the performance of MLP block (MWCapsuleNets) is better than that of conv block (CWCapsuleNets). In the three-class classification task, our reference baseline Mousavi and Afghah (2019) only used a combination of convolutional network and seq2seq, and used SMOTE to balance the data, which can make the sensitivity of class V reach 100%. Therefore, it is reasonable that our model can make sensitivity of class V achieve 100%. Transformer-based models use a self-attention mechanism for context awareness and has very successful performance in processing sequences.There have been studies to enhance and detect ECG signals (Hu et al., 2022; Meng et al., 2022; Xia et al., 2023). However, transformer does not have any inductive bias and often requires a large amount of data to train to perform better. The purpose of this paper is to resolve the classification of single-lead arrhythmias under sample imbalance, so transformer is not considered for the time being.

Although the MLP + WCapsule + Seq2seq model shows excellent performance, it fails to achieve the expected performance in the classification of class S. Especially in less-sample experiments, as shown in Table 4, there is still a gap between its sensitivity and existing work’s. This may be because when optimizing MLP, the overfitting performance is inhibited by reducing the number of stacked layers. So, the learning performance of MLP fails to reach the optimal effect.

Although our model has achieved good results in arrhythmia classification, it also has the limitations of small sample size and single lead data set. We will further evaluate the dependence of the model on leads. Furthermore, we will also consider combining with the other leads or a random lead. In the future, we will verify our model on a larger 12-lead data set. We will also consider the combination of transformer in tasks with more data or more leads.

## 5 Conclusion

The MLP + WCapsule + Seq2seq method proposed in this work can effectively alleviate the problem of sample imbalance in arrhythmia classification, and obtain good performance. At the same time, this method also shows a potential performance with less sample, which also provides a new reference for ECG classification to solve the problem of sample imbalance.

## Data Availability

The original contributions presented in the study are included in the article/Supplementary Material, further inquiries can be directed to the corresponding authors.
