# Supplementary material for: Inter-patient ECG heartbeat classification for arrhythmia classification: a new approach of multi-layer perceptron with weight capsule and sequence-to-sequence combination
Source: Front Physiol. 2023 Sep 28;14:1247587. doi: 10.3389/fphys.2023.1247587 (PMC10569428; doi:10.3389/fphys.2023.1247587)
Supplement: Supplementary file 1 [file DataSheet1.PDF]

Supplementary Material

1 DATASETS

Table S1. Categories of heartbeats existed in the MIT-BIH database based on AAMI.

Category	Description
F	Fusion of ventricular and normal beat
N	Normal beat
Q	Unclassifiable beat
S	Supraventricular ectopic beats
V	Ventricular ectopic beats

Table S2. Details of training and test sets. DS1_2 and DS1_3 are sample data sets after DS1 is divided into the first half and the second half.

	F	N	Q	S	V
**DS1**	414	45798	7	941	3782
**DS2**	388	44198	7	1836	3217
**DS1_2**	8	22376	1	117	1187
**DS1_3**	406	23422	6	824	2595

2 WEIGHT GENERATION AND DYNAMIC ROUTING

In the weight capsule, the weight generation process is also very important. Output $\dot{s}_j$ produces weight k_j:

$$P_j = \frac{\left\| \sum_i c_{ij} \hat{\mu}_{j|i} \right\|^2}{\sum_j \left\| \sum_i c_{ij} \hat{\mu}_{j|i} \right\|^2} \tag{S1}$$

P_j represents the weight of j weight capsules. $\sum_j P_j = 1$.

$$e_j = -\tau P_j \ln P_j \tag{S2}$$

$$g_j = 1 - e_j \tag{S3}$$

Here, $\tau = 1$. After the weight of the length of the capsule module is nonlinear, the new weight obtained is fixed in the range of [0.5, 1]. The formula reassigns the weight of each output capsule. Since the weights are compressed into a range, k_j is approximately bisected by j .

$$k_j = \frac{g_j}{\sum_j g_j} \tag{S4}$$

Similar to the generation method of k_j, the weight f_{ij} generated by the predicted value $\hat{\dot{u}}_{j|i}$ is:

$$Q_{ij} = \frac{\left\|\hat{\dot{u}}_{j|i}\right\|^2}{\sum_{ij}\left\|\hat{\dot{u}}_{j|i}\right\|^2} \tag{S5}$$

$$E_{ij} = -Q_{ij} \ln Q_{ij} \tag{S6}$$

$$G_{ij} = 1 - E_{ij} \tag{S7}$$

$$f_{ij} = \frac{G_{ij}}{\sum_{ij} G_{ij}} \tag{S8}$$

The final output of f_{ij} is approximately bisected by $i \cdot j$.

The main process of dynamic routing algorithm is as follows:

(1) It is necessary to input the prediction weight capsule $\hat{\dot{u}}_{j|i}$ and the iteration times r of the route. Assign the initial logical value $\dot{b}_{ij}$ to 0.

(2) The logical value $\dot{b}_{ij}$ is multiplied by a weight f_{ij}, and the coupling coefficient is obtained by Softmax function.

(3) The weighted sum of the predicted weight capsules is performed according to the coupling coefficient, and then multiplied by the weight k_j generated by $\dot{s}_j$.

(4) Sum after the weight of the capsule to the output is obtained by the compression function weight capsule, this completes a routing iteration.

(5) The next iteration routing needs to calculate the consistency between the output capsule weight $\dot{v}_j$ and the predicted capsule weight $\hat{\dot{u}}_{j|i}$, and then complete the logical value update by adding the logical value $\dot{b}_{ij}$, and then step and initial routing.

Table S3. Dynamic routing between weight capsules

```
Input ROUTING(û_{j|i}, r, l)
    for all capsule i in layer l and capsule j in layer (l+1): ḃ_{ij} ← 0 .
    for all capsule i in layer l and capsule j in layer (l+1): f_{ij} .
--------------------------------------------------------------------------
    for i iterations do :
        for all capsule i in layer l :
            ċ_{ij} ← (softmax(ḃ_{ij} · f_{ij}))
        for all capsule j in layer (l+1) :
            ṡ_j ← k_j Σ_i ċ_{ij} · û_{j|i}
        for all capsule j in layer (l+1) :
            v̇_j ← S − S(ṡ_j) :
        for all capsule i in layer l and capsule j in layer (l+1) : ḃ_{ij} ← ḃ_{ij} + û_{j|i} · v̇_j
    Return v̇_j
```

3 CONTRASTING MODEL STRUCTURE

In order to better evaluate our approach, we compared different heartbeat feature extraction models under the same equipment and environment and the same Seq2seq classification model as in this work. These model structures include:

Baseline: The input data is passed into the Seq2seq model after three layers of convolution and two layers of pooling, as shown in Figure S1. The first layer structure is composed of 32 one-dimensional convolution filters, whose convolution kernel size is 2, stride is 1, and activation function uses ReLU. Then it passes through a maximum pooling layer with both pool-size and stride of 2. The third layer structure is composed of 64 one-dimensional convolution filters, whose convolution kernel size is 2, stride is 1, and activation function uses ReLU. It then passes through a maximum pooling layer with both pool-size and stride of 2. The last layer structure is composed of 128 one-dimensional convolution filters, whose convolution kernel size is 2, stride is 1, and activation function uses ReLU.

MLP block: The input data is passed into the Seq2seq model only after passing through the MLP block proposed in this work. In the MLP block, units=10 and dropout rate=0.8. See Figure S2.

McNets: The input data is passed into the Seq2seq model after passing through the MLP block and a convolutional layer. In the MLP block, units=10 and dropout rate=0.8. See Figure S3.

M-Baseline: The input data is passed into the baseline model after passing through the MLP block proposed in this work. In the MLP block, units=10 and dropout rate=0.8. See Figure S4.

CapsuleNets: After passing through one convolutional layer, the input data are passed into the ordinary capsule network and the Seq2seq model. The convolution layer is composed of 28 one-dimensional convolution filters, whose convolution kernel size is 2, stride is 1, and activation function uses ReLU. This is shown in Figure S5.

MCapsuleNets: After passing through the MLP block proposed in this work, the input data is passed into the ordinary capsule network and the Seq2seq model. In the MLP block, units=10 and dropout rate=0.8. Unlike CapsuleNets, the one-dimensional convolutional layer is replaced with an MLP block here. This is shown in Figure S6.

WCapsuleNets: After passing through a convolutional layer, the input data is passed into the weight capsule network and Seq2seq model. The convolution layer is composed of 28 one-dimensional convolution filters, whose convolution kernel size is 2, stride is 1, and activation function uses ReLU. As shown in Figure S7, different from CapsuleNets, the ordinary capsule model is replaced with a weighted capsule model here.

CWCapsuleNets: After passing through a convolutional module, the input data is passed into the weight capsule network and Seq2seq model. As shown in Figure S8, the convolutional module replaces the fully connected layer of MLP block in this work with a convolutional layer. This convolution layer is composed of 28 one-dimensional convolution filters, whose convolution kernel size is 2, stride is 1, and activation function uses ReLU.

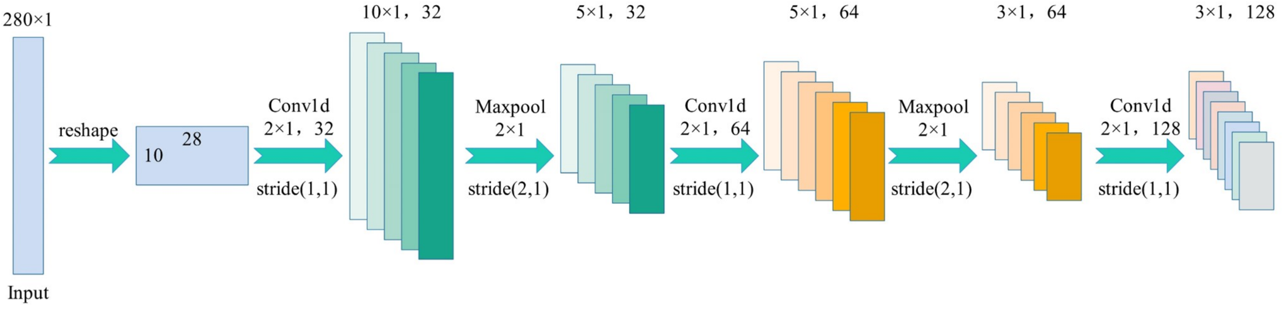

Figure S1. Baseline.

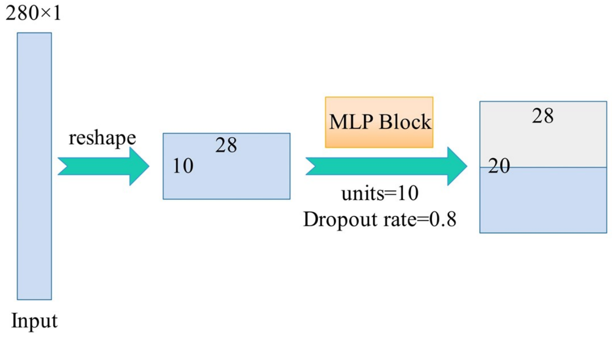

Figure S2. MLP block.

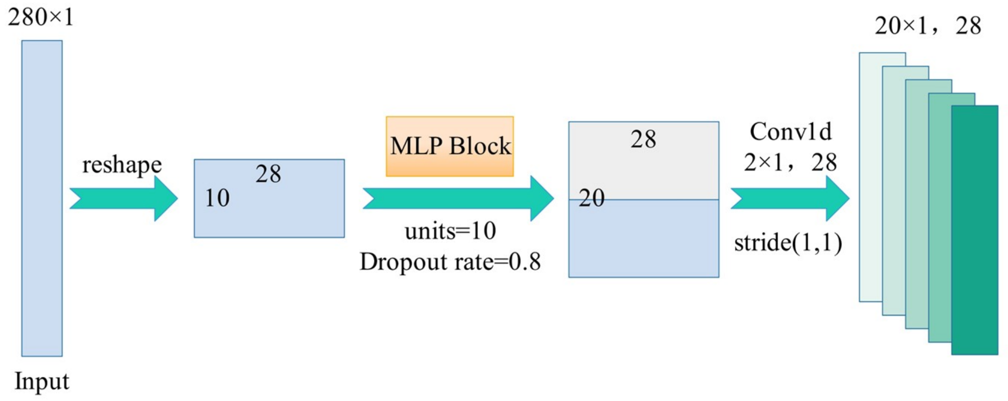

Figure S3. McNets.

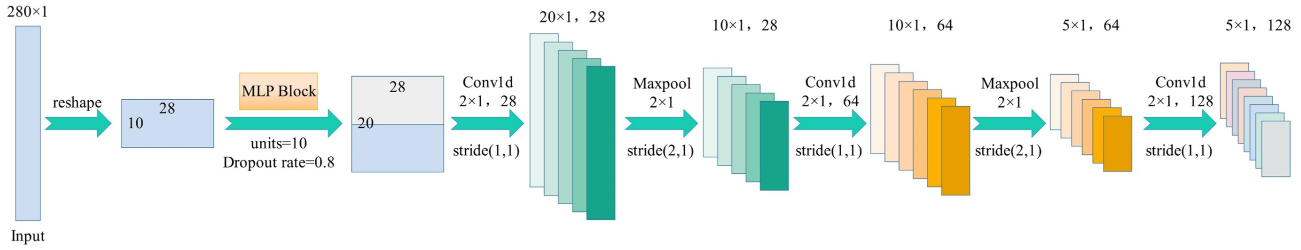

Figure S4. M-Baseline.

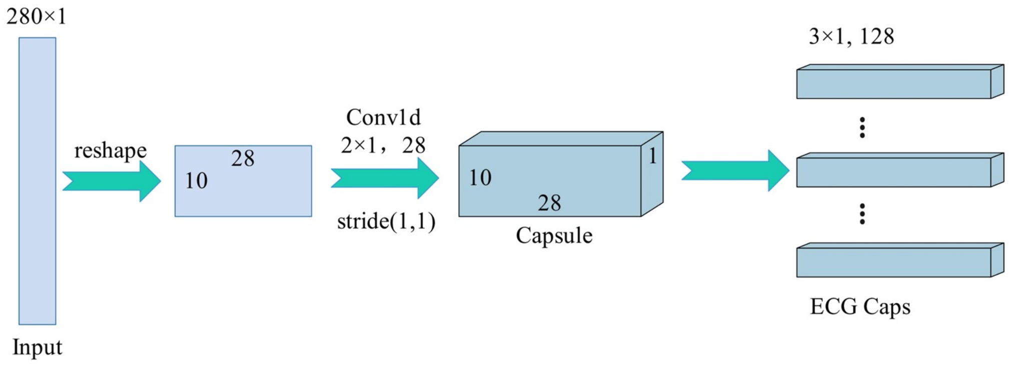

Figure S5. CapsuleNets.

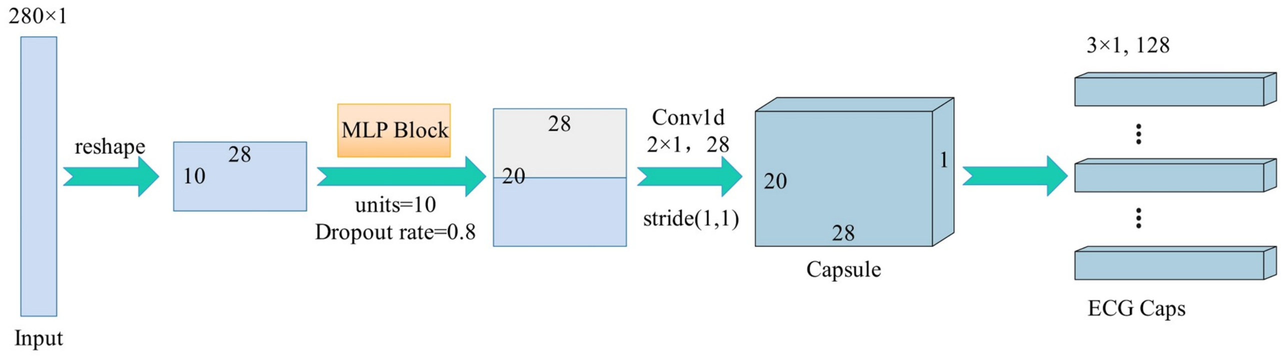

Figure S6. MCapsuleNets.

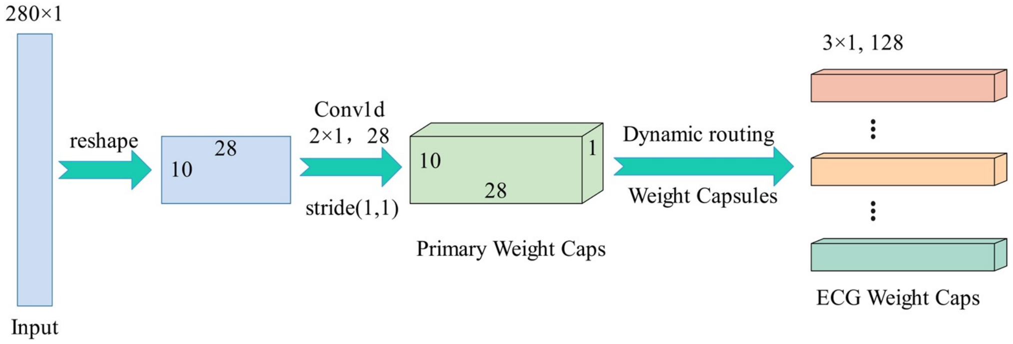

Figure S7. WCapsuleNets.

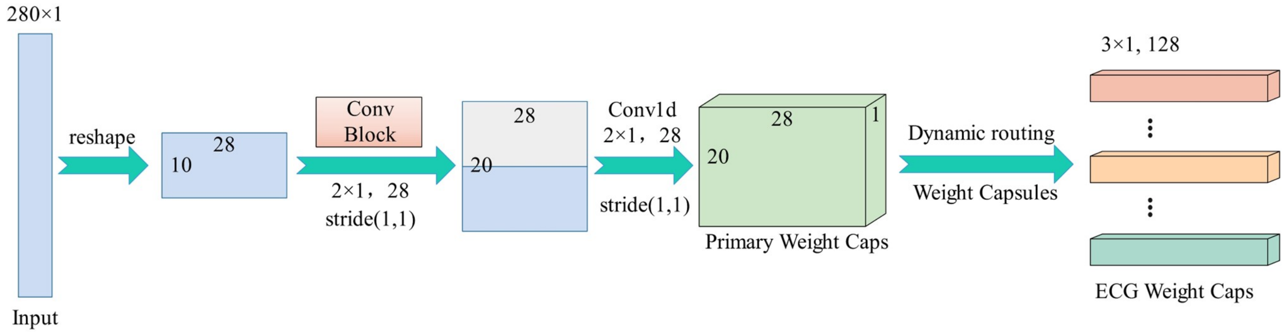

Figure S8. CWCapsuleNets.

4 RESULTS

Table S4. Inter-patient paradigm: The performance of the proposed heartbeat classifier compared with other comparison models, considering DS2 as test dataset based on the MIT-BIH arrhythmia database for the considered groups, N, S, V. SMOTE is used in this experiment.

**Model**	**ACC**	**N**			**S**			**V**		
		**SEN**	**SPEC**	**PPV**	**SEN**	**SPEC**	**PPV**	**SEN**	**SPEC**	**PPV**
Baseline	99.52	99.65	96.12	99.56	89.32	99.67	91.26	99.81	**100**	**99.94**
MLP+Seq2seq	99.70	99.69	98.65	99.85	95.53	99.70	92.61	99.94	99.97	99.57
McNets+Seq2seq	99.67	99.52	99.72	99.97	98.47	99.77	94.41	100	99.74	96.39
M-Baseline	99.51	99.22	99.70	99.97	98.86	99.38	85.98	100	99.88	98.35
CapsuleNets+Seq2seq	99.79	99.72	**99.94**	**99.99**	**99.02**	99.76	94.10	99.69	99.92	98.92
MCapsuleNets+Seq2seq	99.70	99.58	99.56	99.95	98.80	99.63	91.11	99.50	99.95	99.22
CWCapsuleNets+Seq2seq	99.82	99.82	98.97	99.88	96.95	99.83	95.70	100	99.99	99.88
WCapsuleNets+Seq2seq	**99.87**	**99.86**	99.37	99.93	98.04	**99.89**	**97.30**	100	99.97	99.57
MWCapsuleNets+Seq2seq	99.74	99.74	98.54	99.83	95.75	99.80	94.98	100	99.95	99.26
MWCapsuleNets+Seq2seq$^-$	99.83	99.78	99.62	99.96	98.75	99.79	94.82	100	99.99	99.88

[1] The bolded and underlined values are the highest values in this metric.
[2] $^-$ means SMOTE is not used in this part.

Table S5. Inter-patient paradigm: The performance of the proposed heartbeat classifier compared with other comparison models, considering DS2 as test dataset based on the MIT-BIH arrhythmia database for the considered groups, N, S, V, F. SMOTE is used in this experiment.

**Model**	**ACC**	**N**			**S**			**V**			**F**		
		**SEN**	**SPEC**	**PPV**	**SEN**	**SPEC**	**PPV**	**SEN**	**SPEC**	**PPV**	**SEN**	**SPEC**	**PPV**
Baseline	99.04	98.64	89.42	98.69	69.50	98.86	70.07	99.97	99.92	98.89	94.07	99.93	91.02
MLP+Seq2seq	99.85	99.71	99.50	99.94	98.69	99.76	93.98	99.97	99.93	99.04	94.07	99.98	97.59
McNets+Seq2seq	99.55	99.10	97.81	99.73	91.67	99.46	86.75	99.97	99.91	98.74	98.97	99.73	74.13
M-Baseline	98.33	95.51	99.41	99.92	98.31	97.66	61.73	99.97	99.59	94.36	87.89	98.84	37.75
CapsuleNets+Seq2seq	99.17	98.12	97.54	99.69	92.21	99.12	80.05	99.97	99.69	95.71	89.18	99.34	51.56
MCapsuleNets+Seq2seq	99.75	99.41	99.50	99.94	98.31	99.65	91.58	99.97	99.86	97.96	96.65	99.89	87.41
CWCapsuleNets+Seq2seq	99.87	99.72	**99.78**	**99.97**	**99.13**	99.75	93.86	99.97	99.96	99.38	97.42	**99.99**	98.69
WCapsuleNets+Seq2seq	99.90	99.92	99.34	99.92	97.06	99.96	98.89	99.97	99.92	98.92	92.78	99.93	91.37
MWCapsuleNets+Seq2seq	**99.97**	**99.98**	**99.78**	**99.97**	**99.13**	**99.98**	**99.45**	99.97	**99.98**	**99.69**	**99.23**	**99.99**	**98.72**

[1] The bolded and underlined values are the highest values in this metric.

Table S6. Inter-patient paradigm: The performance of the proposed heartbeat classifier compared with other comparison models, considering DS2 as test dataset based on the MIT-BIH arrhythmia database for the considered groups, N, S, V. SMOTE is used in this experiment.

Model	ACC	N			S			V		
		SEN	SPEC	PPV	SEN	SPEC	PPV	SEN	SPEC	PPV
Baseline	97.46	**99.93**	63.63	96.01	0.00	**99.99**	0.00	99.78	99.93	98.98
MLP+Seq2seq	**97.48**	98.60	**79.55**	**97.68**	**43.30**	98.29	49.59	93.78	99.96	99.34
McNets+Seq2seq	97.44	99.91	63.54	95.99	0.00	99.93	0.00	99.69	99.98	99.66
M-Baseline	97.14	98.74	72.76	96.94	36.98	99.02	**59.35**	87.66	99.41	91.23
CapsuleNets+Seq2seq	96.91	98.96	64.92	96.10	2.89	99.31	13.87	98.88	99.61	94.70
MCapsuleNets+Seq2seq	**97.48**	99.91	64.40	96.09	0.38	99.98	46.67	**100**	88.00	98.35
CWCapsuleNets+Seq2seq	97.09	99.22	67.83	96.44	10.90	98.91	27.75	94.64	99.98	99.74
WCapsuleNets+Seq2seq	97.12	98.88	68.17	96.45	12.20	98.92	30.39	99.44	**99.99**	**99.88**
MWCapsuleNets+Seq2seq	96.83	98.47	67.30	96.34	9.80	98.55	20.81	99.69	**99.99**	**99.88**
Baseline*	97.45	99.91	63.54	95.99	0.00	**99.96**	0.00	99.81	99.95	99.35
MLP+Seq2seq*	97.11	99.39	65.06	96.13	5.99	99.22	22.87	95.68	**100**	**100**
McNets+Seq2seq*	97.48	**99.94**	63.74	96.01	0.00	99.95	0.00	**100**	99.99	99.88
M-Baseline*	94.41	94.07	73.67	96.90	27.56	94.12	15.37	94.50	99.98	99.67
CapsuleNets+Seq2seq*	**97.50**	99.86	64.59	96.12	2.20	99.87	**40.00**	99.69	99.99	99.88
MCapsuleNets+Seq2seq*	48.79	14.77	**98.02**	**98.49**	**94.30**	20.45	4.37	98.45	99.99	99.87
CWCapsuleNets+Seq2seq*	97.10	99.09	66.19	96.24	6.70	99.15	23.38	99.19	99.93	99.07
WCapsuleNets+Seq2seq*	97.14	99.32	64.39	96.06	1.80	99.36	9.82	99.88	99.99	99.81
MWCapsuleNets+Seq2seq*	97.33	99.66	64.03	96.03	0.82	99.69	9.15	**100**	99.99	99.88

[1] * means DS1_2 is the training set, with SMOTE, the bolded values are the highest values in this metric.
[2] Without * means DS1_2 is the training set, without SMOTE, the bolded and underlined values are the highest values in this metric.

Table S7. Inter-patient paradigm: The performance of the proposed heartbeat classifier with different dropout rate, considering DS1_3 as training set and DS2 as test dataset based on the MIT-BIH arrhythmia database for the considered groups, N, S, V, F. SMOTE is not used in this experiment.

Model	Dropout Rate	ACC	N			S			V			F		
			SEN	SPEC	PPV	SEN	SPEC	PPV	SEN	SPEC	PPV	SEN	SPEC	PPV
MWCapsuleNets+Seq2seq	0.4	99.46	99.38	**97.04**	**99.63**	81.21	99.56	87.71	**99.97**	99.86	98.05	87.11	99.51	58.38
	0.6	98.90	97.88	93.20	99.15	79.58	97.95	59.88	99.28	**99.97**	**99.59**	92.27	99.99	98.88
	0.7	99.22	99.07	95.43	99.44	73.86	99.30	80.28	99.50	99.85	97.88	88.14	99.37	52.37
	0.8	**99.75**	**99.95**	95.55	99.45	**86.87**	**99.91**	**97.37**	**99.97**	99.94	99.07	88.14	**100**	**100**
	0.9	99.56	99.57	95.01	99.39	82.79	99.64	89.94	**99.97**	99.82	97.41	**93.04**	99.97	95.50

[1] The bolded and underlined values are the highest values in this metric.

5 EXPERIMENTAL RESULTS

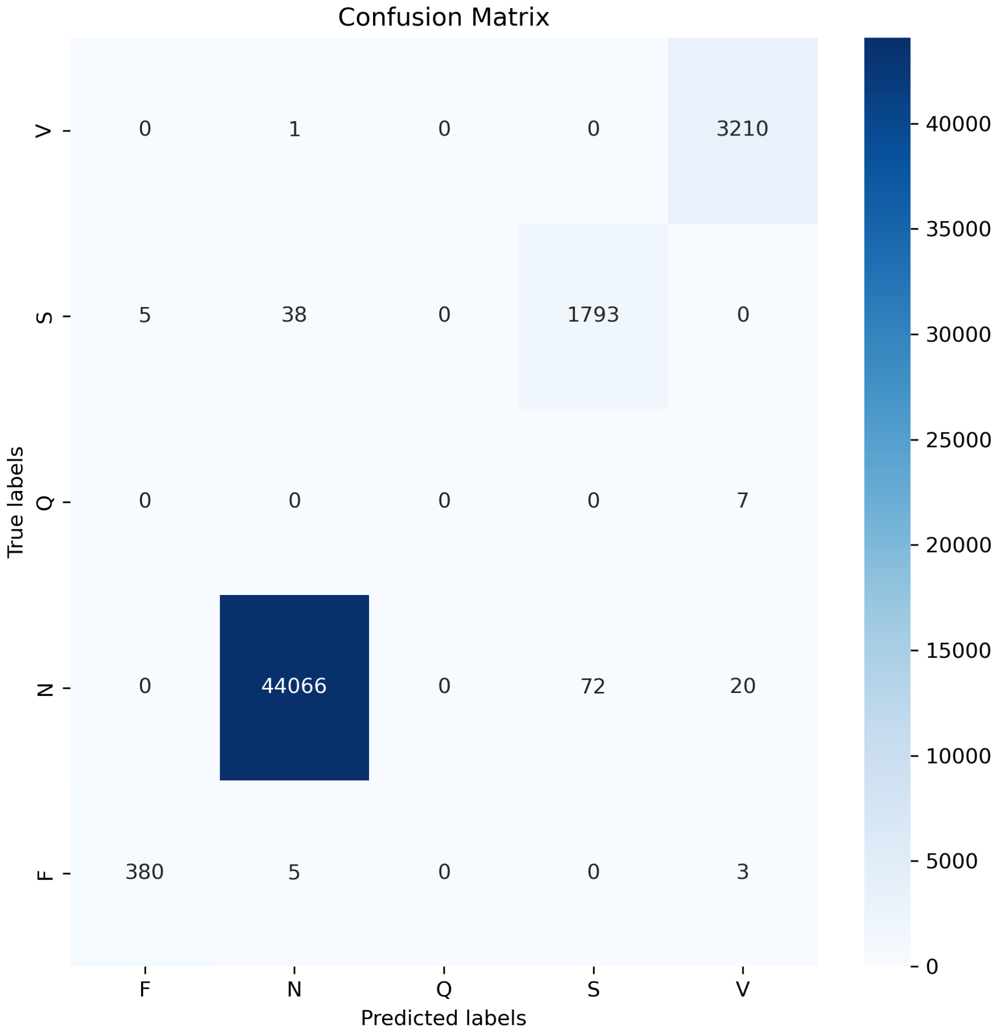

Figure S9. Confusion matrix of MWCapsuleNets in five-classification tasks without SMOTE and using DS1 as the training set.

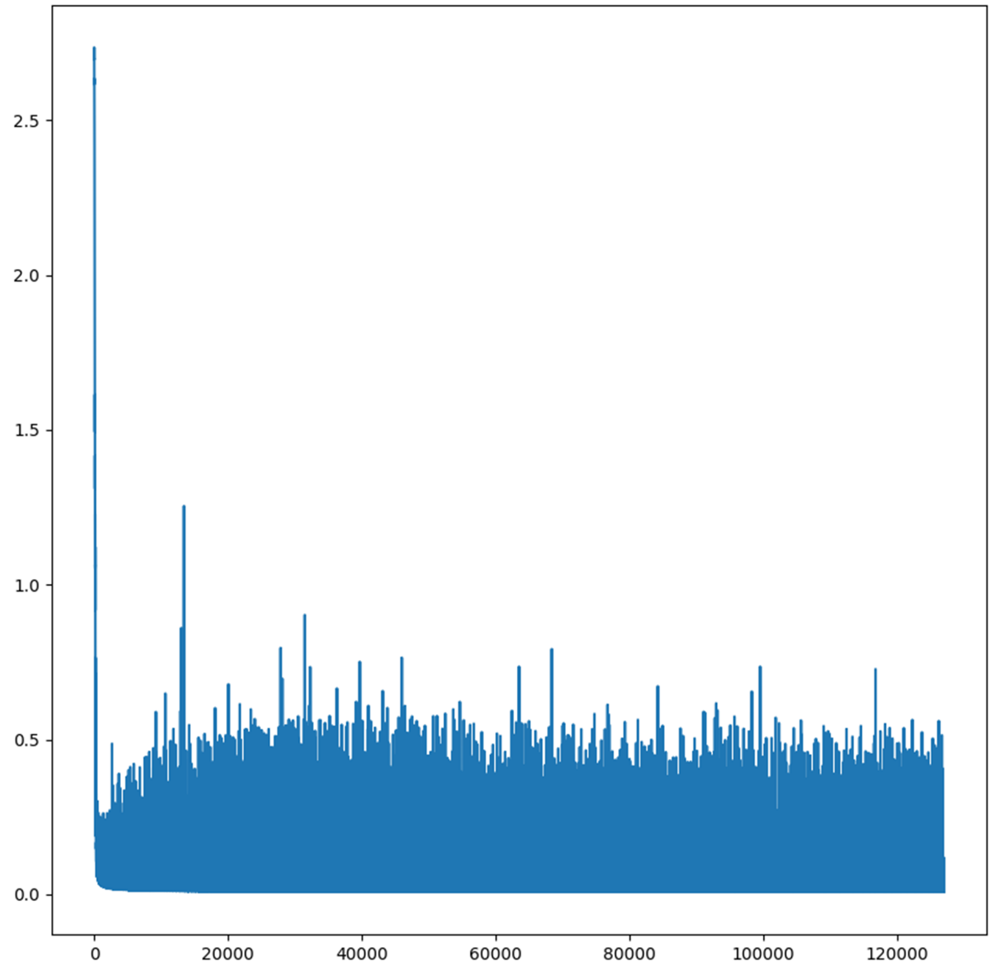

Figure S10. Loss of MWCapsuleNets in five-classification tasks without SMOTE and using DS1 as the training set.

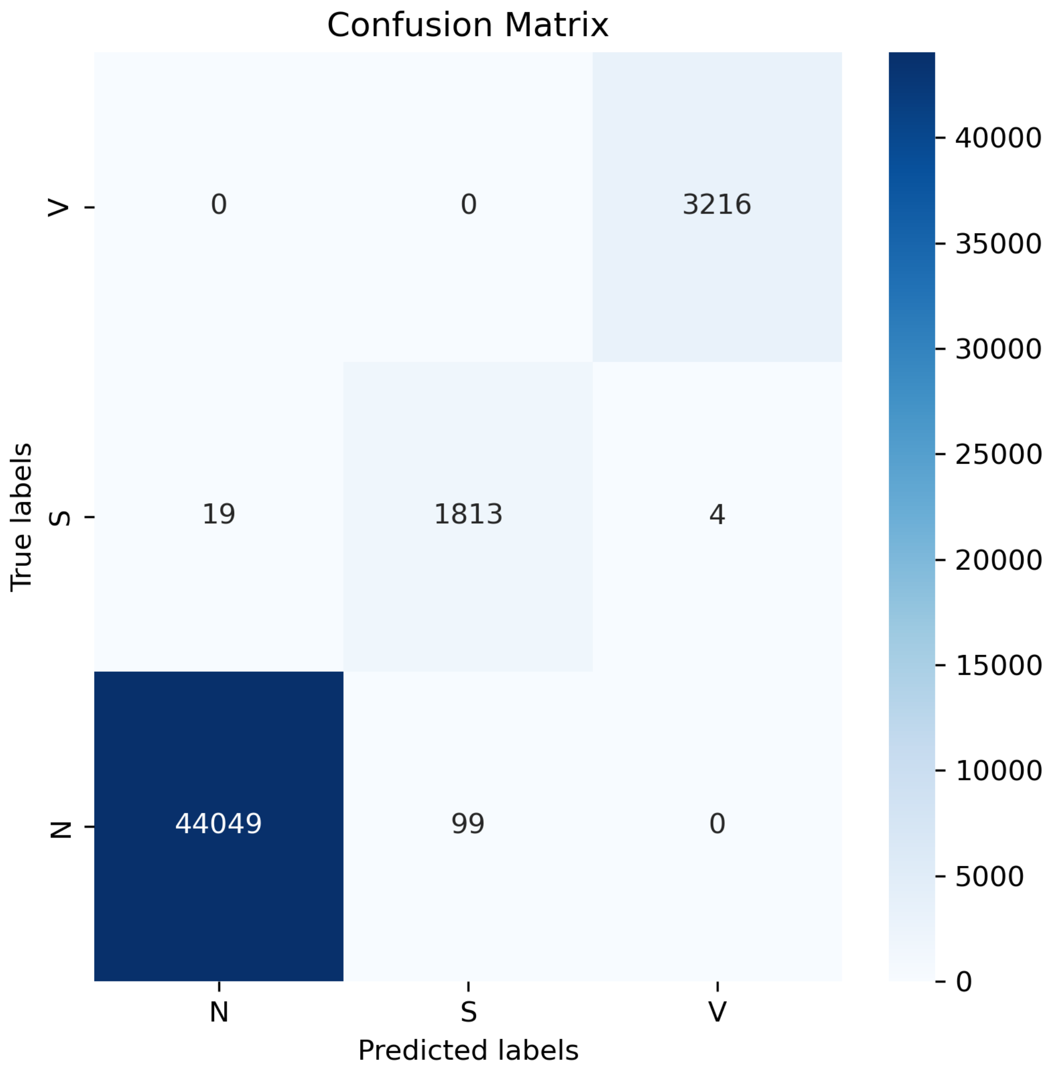

Figure S11. Confusion matrix of MWCapsuleNets in three-classification tasks without SMOTE and using DS1 as the training set.

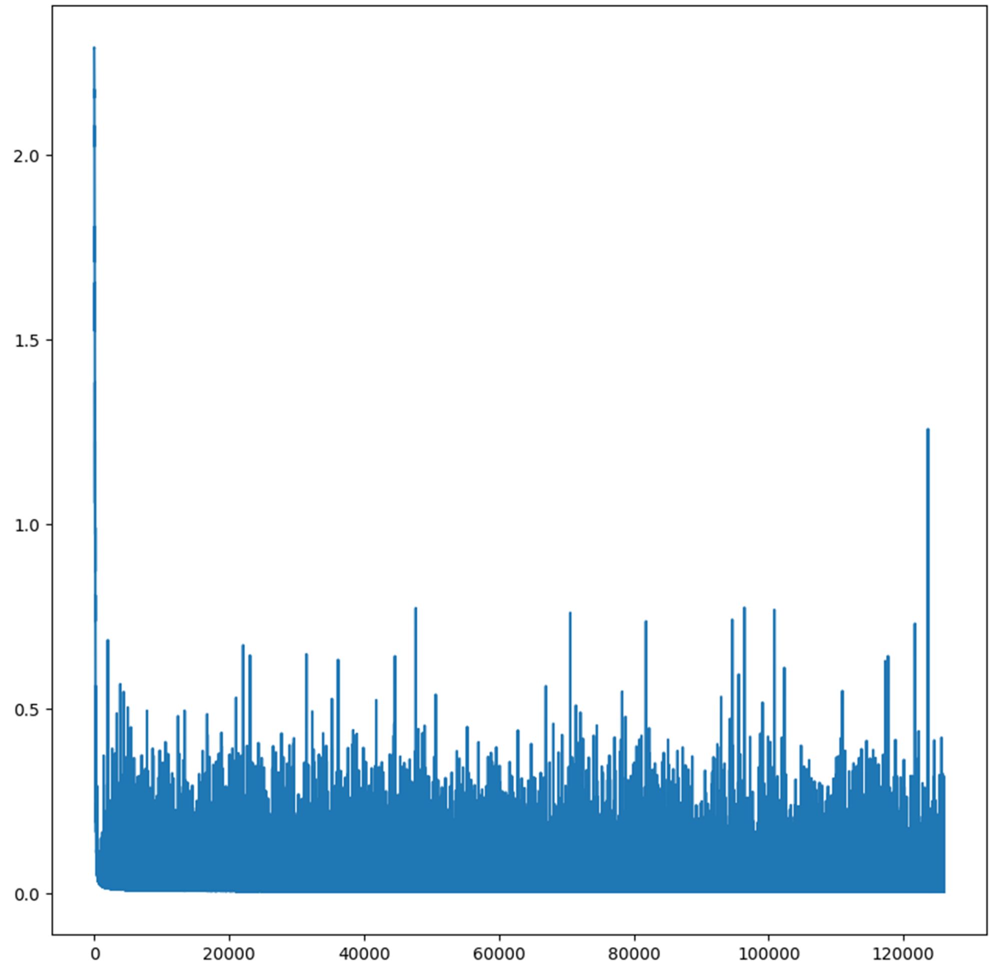

Figure S12. Loss of MWCapsuleNets in three-classification tasks without SMOTE and using DS1 as the training set.
